# The combination of a medial pivot design with kinematic alignment principles in total knee arthroplasty can ensure a closer to normal knee kinematics than combining mechanical alignment and more traditional implant designs: An umbrella review

**DOI:** 10.1002/jeo2.70358

**Published:** 2025-07-18

**Authors:** Pier F. Indelli, Bruno Violante, Pawel Skowronek, Marko Ostojić, Nicolas Bouguennec, Giuseppe Aloisi, Christian Schaller, Umile Giuseppe Longo

**Affiliations:** ^1^ European Society of Sports Traumatology, Knee Surgery Arthroscopy (ESSKA)–European Knee Associates (EKA) Board Luxembourg Luxembourg; ^2^ European Society of Sports Traumatology, Knee Surgery Arthroscopy (ESSKA) Basic Science Committee Luxembourg Luxembourg; ^3^ Paracelsus Medical University (PMU), Institute of Biomechanics Paracelsus Medical University Salzburg Austria; ^4^ Paolo Aglietti Gait Lab, CESAT, Azienda Sanitaria Toscana Centro Fucecchio Italy; ^5^ Orthopaedic and Trauma Department Żeromski Specialist Hospital Krakow Poland; ^6^ Dipartimento di Medicina Clinica, Sanita’ Pubblica, Scienze della Vita e dell'Ambiente Universita’ degli Studi dell'Aquila L'Aquila Italy; ^7^ Department of Medicine and Surgery, Fondazione Policlinico Universitario Campus Bio‐Medico and Research Unit of Orthopaedic and Trauma Surgery Università Campus Bio‐Medico di Roma Roma Italy

**Keywords:** gait analysis, kinematic alignment, knee, medial pivot, review, TKA

## Abstract

**Purpose:**

The recent introduction of personalized alignment strategies in total knee arthroplasty (TKA) has transformed adult reconstruction. Proponents advocate for these techniques due to their kinematic benefits compared to traditional methods. Current literature supports combining medial‐pivot designs with kinematic alignment (KA) surgery. This review summarizes the application of gait analysis in KA medial‐pivot TKA and recommends gait parameters related to patient satisfaction.

**Methods:**

This review followed the Preferred Reporting Items for Systematic Reviews and Meta‐Analyses extension for Scoping Reviews (PRISMA‐ScR). One hundred twenty‐one articles from the three search engines underwent a preliminary title/abstract and full‐text screening. The final screening resulted in 23 systematic reviews (SR), meta‐analyses (M‐A) and narrative reviews (NR) as core articles of the current umbrella review.

**Results:**

Out of the original 121 SR/M‐A/NR articles, 23 (19%) were ultimately evaluated based on the reported results. Twelve articles fell into the first category (gait analysis following TKA as the main topic), five articles were designated for the second category (knee implant design), only one article was classified in the third category (kinematic alignment) and five articles were assigned to the fourth category (a combination of all main topics).

**Conclusions:**

The literature investigating the relationship between kinematic and spatiotemporal data and clinical outcomes following KA medial pivot TKA is limited. Few studies included in the current review showed that remote measurements using wearable sensors are more informative than patients' reported outcome measurements (PROMs) regarding a patient's daily level of activities and, ultimately, gait. The current review demonstrated that combining KA and MP designs can ensure a knee kinematic closer to normal than combining MA and more traditional implant designs.

**Level of Evidence:**

Level I.

**Abbreviations**
CR: posterior cruciate retention
GR: general review
GRF: ground reaction force
KA: Kinematic alignment
MA: mechanical alignment
M‐A: meta‐analysis
MP: medial pivot
NR: narrative review
OA: osteoarthritis
PROMs: patients' reported outcome measurements
PS: posterior cruciate substitution
rKA: restricted kinematic alignment
SR: systematic reviews
TKA: total knee arthroplasty
UKA: unicompartmental knee arthroplasty
uKA: unrestricted kinematic alignment

## INTRODUCTION

Total knee arthroplasty (TKA) surgery has recently shifted from a traditional ‘one size fits all’ approach towards a more personalized strategy catering to individual needs [16]. Regarding surgical techniques, various lower limb alignments have been introduced as alternatives to conventional mechanical alignment (MA), aimed at restoring the native ‘functional’ kinematics and natural perception of the knee. These new techniques range from resurfacing the knee without limitations in postoperative alignment (unrestricted kinematic alignment or uKA) to imposing restrictions by adjusting the alignment to predetermined safe zone criteria (restricted KA or rKA), ultimately striving to restore native ligament laxities for ligament isometry (functional alignment or FA) [6, 37, 48]. This ‘kinematic approach’ focuses on aligning the prosthetic components to mimic the patient's native joint anatomy and function instead of adhering to fixed alignment principles [35]. On the implant design side, medial pivot (MP) implant designs are recently growing in popularity worldwide [18] because of the theoretical advantage of increasing sagittal plane stability and replicating a few characteristics of native joint kinematics [32, 40].

Much is known about the biomechanics of the native knee and the end‐stage knee osteoarthritis patient. Initially performed through static images, gait analysis has recently evolved due to 3D motion capture technologies. This approach, which uses infrared camera‐based systems and skin markers to reconstruct anatomical segments and is sometimes paired with dynamic EMGs, has been utilized to analyze the gait cycle in patients who have undergone total knee arthroplasty (TKA) based on mechanical alignment surgical principles [5]. These patients showed altered biomechanics in their lower extremities, but the impact of these alterations on patient satisfaction remains unclear [3].

While significant improvements in patient‐reported outcome measurements (PROMs) or subjective knee function following KA TKAs have been reported after comparison with MA TKAs [36], there is a profound lack of knowledge on the impact of new TKA alignment philosophies on postoperative knee kinematics as evaluated by modern gait analysis. Despite MP TKA implants being recently advocated as ideal designs to be combined with KA TKA [10], few basic science studies have evaluated the triple combination KA‐MP‐gait analysis.

This review aimed to answer the question: What is currently known about gait patterns following KA with MP designs? The authors reviewed systematic reviews (SR), meta‐analyses (M‐A), and narrative reviews (NR) articles published on this topic. Another aim was to examine articles reporting on innovative technologies for three‐dimensional kinematic gait measurements to evaluate the combined effects of KA‐MP on gait analysis.

## MATERIALS AND METHODS

The local IRB approved this umbrella review as part of an ongoing gait analysis study in an institutional TKA research project (IRB: SABES 71/2023 and 17/2024). The Preferred Reporting Items for Systematic Reviews (PRISMA‐ScR) recommendations were strictly followed (Supporting Information S1: Table 1). First, the International Prospective Register for Systematic Reviews (PROSPERO) database was searched to explore the presence of similar studies (53 ongoing reviews on TKA and GAIT were identified). The authors searched for reviews registered on PROSPERO but did not register their protocol. The electronic search was done in January 2025 on PubMed, Embase, and Epistemonikos.

Inclusion criteria were any review (SR, M‐A, scoping reviews and narrative reviews) that reported on gait analysis following kinematic alignment TKA with a medial pivot design and no restriction on publication date. Exclusion criteria were clinical studies (RCT, cohort, case‐control, case series, case reports, technical notes, editorials or letters to the editor), reviews that pooled results for all types of implants and alignment techniques without stratifying results, and reviews in languages other than English.

**Figure 1:**
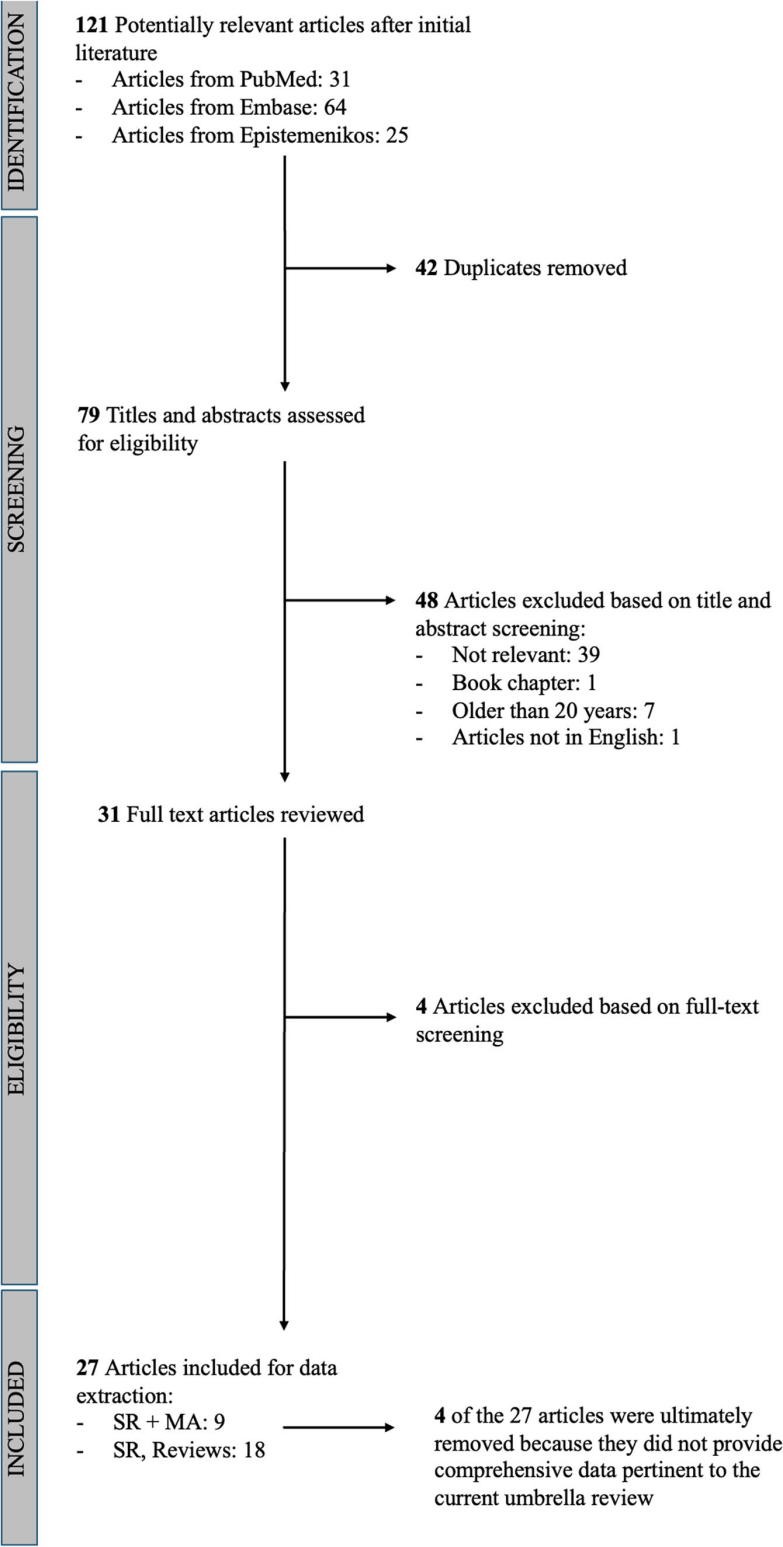
PRISMA flow diagram showing the study screening process.

The search terms included: #1: Gait [Title/Abstract] AND analysis [Title/Abstract] AND Knee [All]; #2: ‘total knee arthroplasty’ [Title/Abstract] OR ‘total knee replacement’ [Title/Abstract] OR TKA [Title/Abstract] OR TKR [Title/Abstract]; #3: ‘meta‐analysis’ [Title/Abstract] OR ‘metanalysis’ [Title/Abstract] OR review [Title/Abstract]; #4: #1 AND #2 AND #3. The search was considered concluded when no new articles were identified during search field variation. The first screening resulted in 121 articles. All articles were then screened by title and abstract for eligibility by two designed authors. This final screening resulted in a preliminary list of 27 articles: 9 SR/M‐A and 18 SR/NR (Figure 1). This first list of SR/M‐A/NR articles was then screened with the intent of highlighting the study type, the number of articles included, the main topic, the presence of a control group, and the kind of outcome considered (functional or according to gait parameters) (Table 1). After this preliminary screening, the 27 articles underwent a second full‐text screening to include each article in one of four categories to satisfy the main and the secondary purposes of this umbrella review: (1) SR/M‐A/NR articles having gait analysis as a main topic; (2) SR/M‐A/NR articles having knee implant design as a main topic; (3) SR/M‐A/NR articles having KA as a main topic; (4) SR/M‐A/NR articles combining 1 and/or 2 and/or 3. Four of the 27 SR/M‐A/NR articles were ultimately removed because they did not provide comprehensive data pertinent to the current umbrella review (Table 1). The authors ultimately included 23 SR/M‐A/NR articles as core articles of the current umbrella review. Any disagreement was resolved through discussion and consensus between the two reviewers.

**Table 1:** This table includes the 27 articles (SR, M‐A, scoping reviews and NR) that entered the final screening.

Author (year)	Country	Journal	Study type	Articles	Study	Control	Outcomes Functional	Outcomes Gait	Notes
Dong et al. (2023) [9]	China	*Gait & Posture*	SR + MA	13 (369 knees)	TKA	UKA	N/A	x	TKA vs UKA comparison
Kakoulidis et al. (2023) [19]	Greece	*KSSTA*	SR + MA	15 (1158 TKA)	MP‐TKA	PS‐TKA	X	x	Medial pivot TKA vs PS‐TKA comparison
Van Essen et al. (2023) [47]	Australia	*The Knee*	SR + MA	26 (2776 TKA)	KA‐TKA	MA‐TKA	X	x	KA‐TKA vs MA‐TKA comparison
Feng et al. (2023) [11]	China	*Arthroplasty*	SR	25 (823 TKA)	Wearable sensors for gait analysis after TKA	N/A	X	x	Analysis of applications of wearable sensors for gait analysis after TKA
Gianzina et al. (2023) [12]	Greece	*The Knee*	SR	12 (474 UKA/TKA)	pre‐KA post‐KA	N/A	X	x	Evaluation of change/recovery in gait function pre‐ and post‐ TKA
Risitano et al. (2023) [33]	Italy/USA	*Arthroplasty*	SR	9 (197MP/192 PS)	MP‐TKA PS‐TKA	N/A	X	x	Medial pivot TKA vs PS‐TKA comparison
Vij et al. (2022) [50]	USA	*J Clin Orthop Trauma*	SR	35	N/A	N/A	N/A	x	Uses of gait analysis in TKA
Li et al. (2022) [23]	China	*J Orthop Surg Res*	SR + MA	9 (351 TKA)	CR‐TKA	CS‐TKA	X	x	CR‐TKA vs CS‐TKA comparison
Van Criekinge et al. (2022) [45]	Belgium	*Gait & Posture*	SR + MA	24 (1'178pts)	TKA/UKA	healthy knees	X	N/A	Trunk control: comparison TKA vs “normal”
Bragonzoni et al. (2019) [4]	Italy	*Gait & Posture*	SR	13	pre‐TKA	Post‐TKA	X	N/A	Evaluation knee proprioception pre‐ and post‐TKA
di Laura Frattura et al. (2018) [7]	Switzerland/Italy	*J Orthop*	SR	11 (1237 TKA)	Risk of Fall in TKA	N/A	X	N/A	Risk factors for falls pre‐ and post‐ TKA
van der Straaten et al. (2018) [46]	Belgium	*Gait & Posture*	SR	23 (5 TKA/18 healthy)	TKA	healthy knees	N/A	x	Mobile kinematic assessment in TKA vs healthy knees
Domínguez‐Navarro et al. (2018) [8]	Spain	*Gait & Posture*	SR + MA	8 (567 TKA/THA)	balance training after TKA/THA	“normal” training after TKA/THA	X	x	Proprioceptive and balance training vs normal training after TKA or THA
Nha et al. (2018) [28]	South Korea	*PLoS One*	SR + MA	7 (99 TKA/82 UKA)	TKA	UKA	N/A	x	TKA vs UKA comparison during level walking
Papagiannis et al. (2018) [31]	Greece	*J Orthop*	Review	na	N/A	N/A	N/A	x	Narrative review on gait analysis methodology
Vaienti et al. (2017) [43]	Italy	*Acta bio‐medica*	Review	na	N/A	N/A	N/A	N/A	Narrative review on knee and TKA
Tan et al. (2016) [42]	Australia	*Gait & Posture*	SR	17	Masai Barefoot Technology	healthy knees	N/A	x	Single 1 paper on TKA
Komnik et al. (2015) [22]	Germany	*Gait & Posture*	SR + MA	35	pre‐KA	post‐KA	X	x	Motion analysis pre and post TKA during ADLs
Harvey et al. (2014) [14]	Australia	*Cochr Dat of SR*	Review	24 (1445 TKA)	N/A	N/A	N/A	N/A	Use of Continuous Passive Motion (CPM) after TKA
Bischoff et al. (2014) [2]	USA/CH	*J Biomechanical Eng*	Review	N/A	N/A	N/A	N/A	N/A	Narrative review on biomechanical analysis in orthopaedics
Schache et al. (2014) [39]	Australia	*Knee*	SR	15	TKA	healthy knees	N/A	x	Lower limb strength after TKA
Naal et al. (2010) [27]	Switzerland	*CORR*	SR	26 (2460 TJA)	Activeness of patients after TJA	N/A	N/A	x	Patient activities after TJA
Ornetti et al. (2010) [29]	France	*Joint Bone Spine*	SR	30	Gait analysis in hip or knee with OA	N/A	N/A	N/A	Gait analysis as a quantifiable outcome for hip or knee OA
Smith et al. (2010) [41]	UK	*Acta Orthop Bel*	SR	3 (247 TKA)	flexion wound	extension wound	X	N/A	Wound closure in flexion vs extension after TKA
McClelland et al. (2007) [25]	Australia	*Knee*	SR	11	TKA	healthy knees	N/A	x	Comparison of TKA vs healthy knees
Marino et al. (2024) [24]	Italy	*Gait & Posture*	SR + MA	28	TKA	healthy knees	N/A	x	Comparison of TKA vs healthy knees
OHTAS (2005) [26]	Canada	*Ont Health Technol Assess Ser*	Report	12	N/A	N/A	N/A	N/A	Physiotherapy rehabilitation after THA/TKA

## RESULTS

Of the original 121 SR/M‐A/GR articles, 23 (19%) were evaluated based on the reported results. Eleven articles fell into the first category (gait analysis following TKA as a main topic), five articles were designated the second category (knee implant design as a main topic), only one article was classified in the third category (kinematic alignment as a main topic), and five articles were assigned to the fourth category (a combination of all main topics).

### Gait analysis following TKA

A total of 12 articles examined gait analysis following TKA (Table 2). Five articles compared the gait parameters of patients who underwent TKA to those with ‘healthy or normal’ knees [8, 24, 25, 45, 46]. The most relevant systematic review for the current umbrella review was authored by McClelland et al. [25], who were among the first to demonstrate that TKA patients experienced a reduced range of motion (ROM), particularly in the swing phase of the gait cycle, compared to healthy controls. In a related study examining spinal mobility, Van Criekinge et al. [45] found that significant differences in trunk motion persisted after TKA, indicating the need for a trunk motion and gait‐retraining rehabilitative protocol to restore postural alignment post‐TKA. Dominguez‐Navarro et al. [8] also advocated balance training protocols alongside conventional physiotherapy to enhance proprioception and functionality after TKA. Van der Straaten et al. [46] reviewed literature relevant to using knee and full‐body sensor systems to assess the kinematics of the knee and lower extremities in TKA patients and healthy controls, suggesting the potential application of inertial sensor systems, accelerometers and gyroscopes to evaluate patients following TKA. Marino et al. [24] confirmed profound differences in kinematic (reduced speed, lower stride length, slower cadence, more extended stance phase) and spatial‐temporal parameters between TKA patients and age‐matched healthy subjects.

**Table 2:** Twelve articles were included in the category “gait analysis following TKA” as a main topic.

Author (year)	Country	Journal	Study type	Articles	Study	Control	Outcomes Functional	Outcomes Gait	Conclusions
Feng et al. (2023) [11]	China	*Arthroplasty*	SR	25	Wearable sensors for gait analysis after TKA	No	x	x	Wearable sensors were able to detect and partially quantify the joint torque and also estimate the intra‐articular load
Gianzina et al. (2023) [12]	Greece	*The Knee*	SR	12	pre‐TKAcpost‐TKA	No	x	x	Accelerometers accurately assess spatiotemporal gait characteristics
Vij et al. (2022) [50]	USA	*J Clin Orthop Trauma*	SR	35	N/A	N/A	N/A	x	Optimal motion analysis parameters still need to be determined
Van Criekinge et al. (2022) [45]	Belgium	*Gait & Posture*	SR + MA	24	TKA/UKA	Healthy knees	x		Need for a trunk motion and gait‐retraining rehabilitative protocol to regain postural alignment after TKA
van der Straaten et al. (2018) [46]	Belgium	*Gait & Posture*	SR	23	TKA	Healthy knees	N/A	x	Support for application of inertial sensor system, accelerometers and gyroscopes to evaluate TKA patients
Domínguez‐Navarro et al. (2018) [8]	Spain	*Gait & Posture*	SR + MA	8	Balance training after TKA	Yes	x	x	Balance training protocols recommended to improve proprioception and functionality after TKA
Komnik et al. (2015) [22]	Germany	*Gait & Posture*	SR + MA	35	pre‐TKA	post‐KA	x	x	Future research needed to obtain tridimensional and inverse‐dynamic data to investigate muscle function following TKA
Schache et al. (2014) [39]	Australia	*Knee*	SR	15	TKA	Healthy knees	N/A	x	TKA patients commonly showed evidence of quadriceps and hamstring weakness following surgery
Naal et al. (2010) [27]	Switzerland	*CORR*	SR	26	Patients’ activities post TKA	N/A	N/A	x	TKA patients still less active than recommended to achieve health‐enhancing activity levels
Ornetti et al. (2010) [29]	France	*Joint Bone Spine*	SR	30	Gait analysis in OA knees	N/A	N/A	N/A	Psychometric properties of gait analysis applied to OA patients still to be determined
Marino et al. (2024) [24]	Italy	*Gait & Posture*	SR + MA	28	TKA	Healthy knees	N/A	x	Persistence of long‐term gait pattern alterations in patients with TKA compared to age‐matched healthy subjects.
McClelland et al. (2007) [25]	Australia	*Knee*	SR	11	TKA	Healthy knees	N/A	x	TKA patients had a reduced ROM, especially in the swing phase of the gait cycle, when compared to healthy controls

Four other articles reviewed patients' monitoring devices (in the gait lab or from remote) to monitor patients before and after TKA [11, 12, 22, 27].

Komnik et al. [22] reported that the literature on gait analysis following TKA was mainly focused on level walking, sagittal plane kinematics and analysis of knee adduction moments. Those authors [22] recommended future research on all three planes of the knee joint and on inverse‐dynamic techniques to investigate muscle function following TKA. Gianzina et al. [12] and Feng et al. [11] reviewed the results of the application of wearable sensors during remote patient monitoring following TKA, showing that wireless sensor devices (accelerometers) could accurately and safely assess spatiotemporal gait characteristics, negating the need for a more advanced and more classical gait analysis technology. Interestingly, Feng et al. [11] also reported that modern wearable sensors could detect and partially quantify the joint torque and estimate the intra‐articular load, which has been reported as a significant limitation compared to data acquired in a classical gait lab setting. Naal et al. [27], in their review of SR on the use of motion sensors (accelerometers more accurately than pedometers) to monitor patients undergoing total joint arthroplasty, reported that those patients are less active than recommended to achieve health‐enhancing activity levels; however, they still appear more active than patients affected by degenerative joint disease of the knee.

The last three articles examined different aspects of using gait parameters to monitor TKA patients. Schache et al. [39] emphasized that TKA patients often displayed signs of quadriceps and hamstring weakness after the surgical procedures, recommending rehabilitative strategies specifically aimed at strengthening these muscle groups to enhance TKA outcomes ultimately. Vij et al. [50] published a fascinating scoping review to identify the most suitable preoperative motion analysis parameters for which a systematic review to determine reliability and validity may be necessary; notably, the authors found that the literature is limited regarding articles addressing preoperative and postoperative outcomes. Finally, Ornetti et al. [29], in a 15‐year‐old article, assessed the psychometric properties (test–retest reliability, validity, sensitivity and specificity) of gait analysis applied to patients affected by degenerative joint disease of the knee: the authors ultimately questioned the use of kinematic parameters as valuable outcome measures in OA. This concept has been contested by more recent literature [21].

### Influence of the design in TKA: Gait analysis, SR and M‐A

Five articles reviewed gait analysis outcomes to determine the impact of different partial or total knee designs (Table 3).

**Table 3:** Five articles were included in the ‘gait analysis outcomes category to determine the impact of different TKA designs’.

Author (year)	Country	Journal	Study type	Articles	Study	Control	Outcomes Functional	Outcomes Gait	Notes
Dong et al. (2023) [9]	China	*Gait & Posture*	SR + MA	13	TKA	UKA	N/A	x	UKA superior to TKA in multiple spatiotemporal and sagittal kinematic parameters
Kakoulidis et al. (2023) [19]	Greece	*KSSTA*	SR + MA	15	MP‐TKA	PS‐TKA	x	x	Similar ROM and similar mean walking speed between MP and PS TKA patients
Li et al. (2022) [23]	China	*J Orthop Surg Res*	SR + MA	9	CR‐TKA	CS‐TKA	x	x	N major kinematic differences between CR and PS patients but PS patients showed an increased second knee flexion peak respect to CR patients
Nha et al. (2018) [28]	South Korea	*PLoS One*	SR + MA	7	TKA	UKA	N/A	x	No significant differences in GRF, overall kinematics, walking speed, or cadence between UKA and TKA patients
Risitano et al. (2023) [33]	Italy/USA	*Arthroplasty*	SR	9	MP‐TKA PS‐TKA	N/A	x	x	MP‐TKA patients showed higher knee rotational moment and greater tibiofemoral external rotation during ROM.

Two SR/M‐A reviews focused on gait analysis outcomes comparing TKA to unicompartmental knee arthroplasty (UKA) patients [9, 26]. Nha et al. [28], reviewing several SR/M‐A gait analysis studies, highlighted that no significant differences in vertical ground reaction force (GRF), joint moment at stance, overall kinematics, walking speed, or cadence existed between UKA and TKA patients during level walking. A few years later, Dong et al. [9], comparing spatiotemporal, kinematic and kinetic gait characteristics during level walking between TKA and UKA patients, found opposite outcomes: medial UKA designs were superior to TKA designs regarding walking speed, stride length, maximum knee flexion at loading, vertical GRF, knee internal rotational moment and knee extension. Compared to the results of Nha et al. [28], Dong et al. [9] reported inconsistent findings, which were justified because gait characteristics were analyzed more systematically and comprehensively with increased studies and an expanded sample size.

Two other SR/M‐A studies compared medial pivot (MP) and posterior‐stabilized (PS) knee designs regarding gait parameters [19, 29]. Kakoulidis et al. [19] reported a similar range of motion (ROM) and similar mean walking speed between MP and PS TKA designs; on the other hand, differences in the three‐plane kinematic data were not reported. Unlike Kakoulidis et al. [19], Risitano et al. [33], in their systematic review, revealed significant kinematic and kinetic differences between MP and PS TKA at all gait analysis phases; in fact, MP TKA showed substantially higher knee rotational moment and greater tibiofemoral external rotation motion during knee flexion than PS TKA.

Finally, Li et al. [23] compared two‐dimensional gait patterns between posterior cruciate retention (CR) and substitution (PS) designs in TKA: no significant differences were found between CR and PS patients in terms of kinematic gait parameters, knee extension, or walking speed; interestingly, PS patients showed an increased second knee flexion peak compared to CR patients.

### Kinematic alignment and gait analysis

Only one study, by Van Essen et al. [47], was included in the current umbrella review.

This study [47] represented a meta‐analysis of randomized controlled trials and observational studies comparing postoperative ROM and gait analysis in TKA performed according to KA versus MA surgical philosophies. According to multiple gait parameters analyzed (including spatiotemporal parameters, kinetics and kinematics analysis), gait and plantar pressure distribution of KA cohorts more closely represented healthy cohorts: KA also showed a weak association of a decreased knee adduction moment (KAM) compared to MA. Unfortunately, this SR/M‐A study [47] could not include articles reporting three‐dimensional kinematic measures of gait.

### Mixed topics

Five articles (Table 4) were included in this umbrella review because they reported data on multiple topics related to the umbrella review's main topics, despite not showing pure gait analysis parameters.

**Table 4:** Five articles were included in the ‘mixed topics’ category because they reported data on multiple topics related to the umbrella review's main topics, despite not showing pure gait analysis parameters.

Author (year)	Country	Journal	Study type	Articles	Study	Control	Outcomes Functional	Outcomes Gait	Notes
Bragonzoni et al. (2019) [4]	Italy	*Gait & Posture*	SR	13	pre‐TKA	Post‐TKA	x	**N/A**	No consensus on improvement or worsening in proprioception before and after TKA
di Laura Frattura et al. (2018) [7]	Switzerland/Italy	*J Orthop*	SR	11	Risk of fall in TKA	N/A	x	**N/A**	OA patients who underwent TKA remained at high risk of falls after surgery
Harvey et al. (2014) [14]	Australia	*Cochr Dat of SR*	Review	24	CPM post TKA	N/A	**N/A**	**N/A**	CPM use following TKA has no effects on active ROM, pain, function, or quality of life
Smith et al. (2010) [41]	UK	*Acta Orthop Bel*	SR	3	Wound closure in flexion	Wound closure in extension	x	**N/A**	Patients with TKA wounds closed in flexion had greater postop ROM
OHTAS (2005) [26]	Canada	*Ont Health Technol Assess Ser*	Report	12	Rehabilitation after TKA	N/A	N/A	N/A	Home‐based physiotherapy better than inpatient physiotherapy after primary TKA; Pre‐rehabilitation not effective.

Two articles [14, 26] were focused on rehabilitation after TKA. The first article [24] was a health technology policy analysis by the Medical Advisory Secretariat, part of Canada's Ontario Ministry of Health and Long‐Term Care. This report [26] had two main findings related to the current umbrella review: first, home‐based physiotherapy instead of inpatient physiotherapy after primary TKA surgery was recommended; second, an exercise programme beginning 4 to 6 weeks before primary TKA surgery was found ineffective. The second review on rehabilitation was published by Harvey et al. [14] the authors focused on the application of continuous passive motion (CPM) following TKA, reporting that CPM did not have clinically significant effects on active ROM, pain, function, or quality of life to justify its routine use.

Another SR article included in this umbrella review was published by Smith et al. [41] the authors investigated whether TKA wounds should be closed in flexion or extension, ultimately showing that patients with TKA wounds closed in flexion had greater flexion and required less physiotherapy sessions compared to those with wounds closed in full extension.

Di Laura Frattura et al. [7] investigated the incidence of falls in patients with degenerative joint disease in the knee who underwent TKA. Interestingly, TKA patients remained at high risk of falls after surgery. Finally, Bragonzoni et al. [4] reviewed the proprioceptive skills in OA patients before and after TKA. Interestingly, no consensus was found in the literature about improving or worsening proprioception before and after TKA.

## DISCUSSION

The most important finding of the current review was that combining KA and MP designs can ensure knee kinematics that are closer to normal than those achieved by combining MA and more traditional implant designs. The authors aimed to identify studies that report data on using various forms of motion analysis following medial pivot total knee arthroplasty performed according to KA principles. In a time when PROMs serve as the primary driver in the TKA decision‐making process, the authors aimed to emphasize the significance of obtaining objective data following knee arthroplasty [30]. Unfortunately, this umbrella review confirmed limited literature examining the relationship between kinematic and spatiotemporal data and clinical outcomes after KA medial pivot TKA. This limitation is likely because alternative alignments in TKA are a relatively new concept, primarily supported by academic or high‐volume surgeons associated with scientific societies [15, 48].

Considering the etymology, the term ‘kinematic alignment’ (KA) in TKA is founded on the principle that all three kinematic axes of knee motion should be respected after resurfacing the knee with the implant. It would be reasonable to assert that, instead of viewing PROMs as the definitive outcome measures, kinematic and kinetic outcome measures comparing different alignments and TKA designs hold greater significance [33]. The increasing use of modern gait analysis platforms equipped with 3D cameras (38), the application of inertial sensor systems, accelerometers, and gyroscopes [46], and finally, the application of wearable sensors during remote patient monitoring [11] are elevating the opportunities to obtain objective data following TKA. To support this, a few studies [11, 12] included in the current umbrella review showed that remote measurements using wearable sensors may be more informative than PROMs regarding a patient's daily level of activities and, ultimately, gait. Although it is a fascinating alternative to more complex gait analysis platforms, Feng et al. [11] reported that modern wearable sensors provided partial estimates of joint torque and intra‐articular load, which has been identified as a significant limitation when compared to data acquired in a traditional gait lab setting. Unfortunately, the current umbrella review confirmed that the available literature [22] on gait analysis following TKA lacks three‐dimensional data, including data on muscle recruitment and function following TKA. Several authors [1, 31, 38, 50] recently pushed on considering pure spatiotemporal parameters (i.e., knee adduction moment, cadence, stride length and velocity) and pure kinematic parameters (i.e., knee ROM, spine ROM and hips ROM) for integration into composite clinical scores.

This umbrella review acknowledges that objective data support the shift from MA to a more personalized alignment, but many of those studies report data obtained with ‘static’ modalities. A significant criticism of KA was the common misbelief that KA systematically brings the joint line of the tibia into varus, interfering with the natural parallelism between the joint line and the floor during heel strike [17, 49]. Hypothetically, KA, which aims to maintain pre‐arthritic anatomy and respects the soft tissue envelope of the knee, should reproduce a closer‐to‐normal gait than fixed, anatomy‐altering alignments. To confirm this, recent data shows that KA achieves more parallelism to the ground in both the bipedal stance phase during standing and the single‐leg stance during gait [13]. A reason for this kinematic behaviour has been found in the valgus pull on the knee joint line during weight bearing or walking, which shifts the kinematically aligned varus knee to the alignment parallel to the floor [13]. On the other side, implanting a mechanically aligned tibial component led to a valgus position during the stance phase of the gait cycle [20]. The study by van Essen et al. [47] has shown a slight superiority of KA over MA in several functional outcomes but without clinical significance: anyway, TKA implanted following KA philosophy may more closely resemble natural knee kinematics in some studies.

With this shift to restoring knee anatomy and making it as physiological as possible, the choice of the implant has been considered crucial. Historically, a medial pivot (MP) design showed the advantage of rotational stability for replicating the physiological knee motion, with the medial condyle being a ‘center of rotation’ [40]. The review by Risitano et al. [34] revealed the kinematic and kinetic advantages of MP TKA over PS implants.

This umbrella review focuses on the combination of KA with MP TKA design. By inductive reasoning, referring to multiple anatomical studies preimplantation and postimplantation [33, 40], MP TKA should easily comply with KA principles. A study by Kaneda et al. [20] has shown that, on two‐ and three‐dimensional gait characteristics, MP and KA combination in TKA successfully reproduced the medial pivot pattern of knee motion and achieved more significant external rotation of the femur relative to the tibia than MA MP TKA. Few recent studies showed that KA could maximize the primary concept of the medial pivot knee design by enhancing its medial pivot pattern during gait [20, 44].

This umbrella review has several limitations. The current literature search identified 121 SR/M‐A/NR articles. However, the vast majority of these articles did not include all the researched terms (‘gait analysis’, ‘kinematic alignment’ and ‘medial pivot’) in the same title or abstract; as a result, our umbrella review revealed a lack of studies assessing gait patterns in patients who underwent TKA using kinematic alignment techniques. Consequently, the findings of this umbrella review have been extrapolated from articles focused on a single topic and later combined. Furthermore, it reveals significant variability regarding the methodology and quality of the included studies. No quality assessment of the included studies was performed because their designs (systematic review, meta‐analysis and narrative review) were too different for a single tool like AMSTAR (Assessing the Methodological Quality of Systematic Reviews) to be applicable.

## CONCLUSION

The current umbrella review, despite its limitations, demonstrates that combining KA and MP designs can achieve knee kinematics closer to normal than those of MA and more traditional implant designs. During this transitional period between classical alignment principles and new strategies, the authors of this umbrella review support the hypothesis that in vivo kinematic studies are essential for understanding knee motion and providing strong evidence of KA's benefits [30] before shifting to different paradigms. Future prospective studies that yield consistent gait outcomes are necessary to determine whether newer surgical approaches would enhance patient satisfaction and implant survival.

## AUTHOR CONTRIBUTIONS

The authors Pier Francesco Indelli, Bruno Violante, and Marko Ostojić were involved in the conceptualization of the project. The formal analysis of the data included in this ‘umbrella review’ was performed, further reviewed, and finally validated by Pawel Skowronek, Nicolas Bouguennec, Giuseppe Aloisi, Christian Schaller and Umile Giuseppe Longo. All authors commented on, revised, and approved the manuscript drafted by Pier Francesco Indelli.

## CONFLICT OF INTEREST STATEMENT

The authors declare no conflicts of interest.

## ETHICS STATEMENT

The local IRB approved this umbrella review as part of an ongoing gait analysis study in an institutional total knee arthroplasty research project (IRB: SABES 71/2023 and 17/2024). No written consent from participants in the study was necessary.

## Supporting information

PRISMA.

## Data Availability

The data supporting this study's findings are available from the corresponding author upon request.
